# Beyond clinical skills: reconceptualizing public health literacy as a core competency in nursing education-a perspective

**DOI:** 10.3389/fpubh.2026.1845586

**Published:** 2026-05-20

**Authors:** Li-ping Gao, Yong Bing, Xiu-li Yu, Jia-xin Wan, Zhao-ning Xu

**Affiliations:** 1 Department of Central Sterile Supply, The First Hospital of Jilin University, Changchun, China; 2 Department of Intensive Care Unit, The First Hospital of Jilin University, Changchun, China; 3 Department of of Gynecology, The First Hospital of Jilin University, Changchun, China; 4 Patient Services Center, The First Hospital of Jilin University, Changchun, China; 5 Department of Nursing Care, The First Hospital of Jilin University, Changchun, China

**Keywords:** community engagement, competency-based education, core competency, curriculum reform, health equity, nursing education, population health, public health literacy

## Abstract

Contemporary public health challenges—including infectious disease pandemics, rising chronic disease burdens, and persistent health inequities—have expanded nursing practice beyond traditional clinical care. Nevertheless, nursing education remains mostly clinical-centered and lacks adequate integration of population health perspectives. This perspective article argues that nursing education needs a fundamental reconceptualization, positioning public health literacy as a core competency rather than an optional addition. We propose a theoretical framework that integrates epidemiological thinking, health promotion, community engagement, policy sensitivity, and cultural safety across curricula, practice, assessment, and institutional support systems. Grounded in the social ecological model, competency-based education, and critical pedagogy, this framework offers concrete implementation pathways. These include curriculum integration, community-based learning, multidimensional assessment, and faculty development. Although barriers such as curriculum density and faculty training gaps exist, strategies—including policy guidance, demonstration courses, and international collaboration—can facilitate reform. This reconceptualization not only enhances nursing competencies but also reshapes professional identity, ultimately preparing a workforce capable of advancing both clinical excellence and population health.

## 1 Introduction

### 1.1 Public health demands on nursing practice

Twenty-first-century public health challenges—including recurrent infectious disease epidemics and pandemics, the rising burden of non-communicable chronic diseases, and persistent health inequities across populations—have fundamentally reshaped health system operations and expanded the scope of nursing practice beyond traditional clinical care (1, 2). For example, during infectious disease outbreaks, nurses should not only provide direct care to critically ill patients but also perform community-level functions such as population screening, health surveillance, risk communication, and immunization coordination (2, 3). Similarly, the long-term management of chronic diseases requires nurses to assess and address the social, behavioral, and environmental determinants influencing patient health outcomes (4). Moreover, enduring health inequities compel nursing practice to confront the structural factors that lead to differential health outcomes across population groups (5, 6). Consequently, contemporary nurses need not only strong clinical skills but also the ability to identify, analyze, and respond to public health issues from a population-oriented perspective.

### 1.2 Clinical focus of traditional nursing education and public health gaps

Historically, nursing education has adopted a clinical-centered model, with curricula and teaching priorities focused heavily on individual-level diagnosis and treatment, emergency procedures, and hospital-based nursing practices (1). While this traditional approach effectively develops basic clinical skills, its public health limitations have become increasingly evident (7). Most nursing programs lack systematic instruction in epidemiology, social medicine, health promotion, and community health management (8, 9). As a result, students often have insufficient knowledge and underdeveloped cognitive frameworks when facing non-clinical situations—such as population health assessment, health data interpretation, community resource coordination, and cross-sectoral collaboration (9, 10). Such an orientation, which prioritizes individual care while marginalizing population health, cannot adequately prepare nurses for the expanded responsibilities imposed by contemporary public health challenges (11).

### 1.3 Proposed shift: public health literacy as a core competency

Building on the above analysis, this perspective article advances a central thesis: nursing education requires a comprehensive paradigm shift—moving away from the traditional clinical-centered training model toward a framework that positions public health literacy as a core competency. This transformation entails redefining the competency profile of a “qualified nurse” across curriculum design, pedagogical strategies, and assessment criteria. Epidemiological reasoning, health equity awareness, community assessment capabilities, risk communication skills, and intersectoral collaboration should be integrated as central training objectives (12). This proposed reorientation is consistent with evolving international trends in nursing education, where competency-based frameworks increasingly emphasize population health, interprofessional collaboration, and health equity as foundational domains of professional preparation. Only through such a paradigm shift can the nursing profession fully realize its essential functions in disease prevention, health promotion, and population health maintenance amid an increasingly complex public health landscape.

## 2 Public health literacy: conceptual definition and its absence in nursing education

### 2.1 The connotation of public health literacy

Public health literacy refers to the integrated ability of nursing professionals to identify, understand, and address health issues from a population perspective (13). This concept includes foundational knowledge of epidemiology and biostatistics, but also extends across several interrelated dimensions. In terms of attitudes, nurses should adopt prevention-oriented values and a commitment to health equity (9). Regarding social justice, they need to recognize structural factors—such as economic conditions, educational resources, and healthcare access—that shape health outcomes across different populations (9). In community assessment, nurses should comprehensively evaluate community health needs, resource distribution, and risk factors (14). From a prevention orientation, the focus lies on health promotion and risk reduction before disease onset, rather than solely on post-illness treatment (14). Finally, intersectoral collaboration requires nurses to work with non-healthcare sectors—including education, social security, and urban planning—to address the social determinants of health (15). Thus, public health literacy is a comprehensive competency that goes beyond individual care to actively engage with population health.

### 2.2 Complementarity and tension between clinical and public health literacy

In nursing practice, clinical literacy and public health literacy are both complementary and occasionally in tension. Clinical literacy focuses on assessing, diagnosing, treating, and rehabilitating individuals with established illness, emphasizing immediacy, precision, and personalized care. Public health literacy, by contrast, concerns risk identification and prevention among healthy or subclinical populations, prioritizing long-term, population-level, and structural interventions (16). Their complementarity is clear: clinical findings can inform public health strategies, while public health measures—such as immunization and health education—can reduce the clinical burden (17, 18). However, tensions can arise. In resource-limited settings, conflicts may occur between individual emergency care and population-level prevention (16, 17). Moreover, patient-centered clinical reasoning follows a different decision-making logic than population-oriented public health thinking (17). Therefore, nursing education should help students understand and integrate both literacies rather than forcing a choice between them.

### 2.3 Systematic deficiencies in public health literacy development

Despite the growing importance of public health literacy for modern nursing practice, current nursing education systems show systematic deficiencies in developing this competency (19). First, curricula lack systematic instruction, often offering epidemiology, social medicine, and health promotion only as electives or in compressed formats (20). Second, clinical practicums are dominated by hospital rotations, with insufficient time devoted to community-based nursing, limiting exposure to population health interventions (19). Third, assessment systems focus primarily on individual technical skills, with no standardized evaluation of public health competencies such as community assessment or intersectoral communication (19, 21). Finally, many nursing educators lack formal training in public health or community nursing, which constrains their ability to guide students effectively (19, 20). Together, these deficiencies produce graduates with weak knowledge bases, underdeveloped cognitive frameworks, and limited practical skills for addressing population health issues—hindering their capacity to fulfill the expanded roles that contemporary public health contexts demand of nurses.

## 3 Theoretical framework: a restructured nursing education model centered on public health literacy

### 3.1 Components of the model

**Figure 1:**
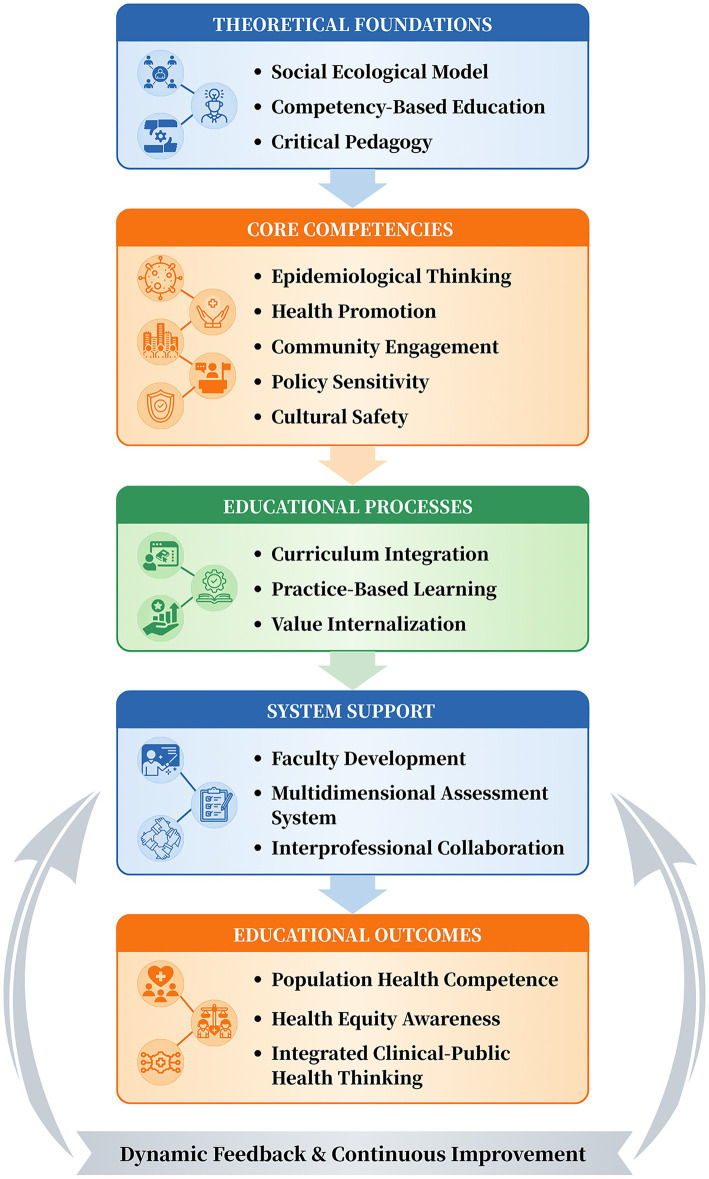
Integrated framework of public health literacy-centered nursing education reform. The figure was created using BioRender.com.

The proposed model for restructuring nursing education centers on public health literacy and includes three interconnected levels. The first level defines core competencies. Epidemiological thinking involves analyzing disease distribution and its contributing factors across populations (22). Health promotion refers to designing interventions that target group behaviors (22, 23). Community engagement means building collaborative relationships to identify and address local health needs (22). Policy sensitivity requires understanding how health policies shape nursing practice and population health (24). Cultural safety involves providing effective care while respecting the cultural backgrounds of those served (24). The second level addresses educational processes through three mechanisms. Curriculum integration systematically embeds public health content across the nursing curriculum rather than isolating it (9). Practice embedding creates authentic population health tasks in clinical and community settings, helping students apply knowledge in action (14). Value internalization uses case discussions and reflective learning to foster professional values centered on prevention and equity. The third level comprises system supports. Faculty development strengthens educators’ public health teaching competencies through targeted training (25). The assessment system uses a multidimensional, formative approach to measure public health literacy (15). An interprofessional collaboration mechanism connects nursing with disciplines such as public health, social work, and urban planning (15). Together, these three levels form a coherent educational restructuring model (Figure 1).

### 3.2 Theoretical foundations of the model

The model draws on three theoretical foundations. First, the social ecological model posits that individual health results from interactions across multiple levels—individual, interpersonal, community, organizational, and policy (26, 27). Nursing education should therefore help students analyze health issues across these levels, not only at the individual or clinical level. Second, competency-based medical education focuses on designing learning objectives around the specific competencies needed for future practice, using formative assessment and feedback to support skill development (28). The present model follows this approach by breaking public health literacy into teachable and assessable competency dimensions. Third, critical pedagogy encourages learners to examine social power structures, the roots of inequality, and their health impacts-thereby becoming agents of change (29). In nursing education, critical pedagogy supports students’ awareness of structural health inequities and their willingness to act.

In addition, the proposed model is further supported by internationally established competency frameworks that reinforce its conceptual robustness and practical relevance. For instance, the American Association of Colleges of Nursing Essentials framework identifies population health as a central domain of nursing competence, underscoring the integration of individual clinical care with broader community and population-level health improvement (30, 31). Similarly, the Consortium of Universities for Global Health Interprofessional Global Health Competencies emphasize cross-sectoral collaboration and global health perspectives, highlighting the importance of interdisciplinary engagement in addressing complex health challenges (32). Collectively, these frameworks provide external validation for the proposed model while offering actionable guidance for translating theoretical constructs into educational practice. They underscore how integrating clinical, behavioral, and systems-level insights fosters more adaptive, responsive, and equitable health education interventions (33). By grounding pedagogy in real-world complexity, the model resists oversimplification and supports learners in navigating ambiguity with critical awareness. This alignment with emergent best practices strengthens its credibility across academic, clinical, and policy domains.

Taken together, these theoretical and framework-based perspectives are mutually reinforcing, providing a comprehensive and coherent foundation for restructuring nursing education around public health literacy.

## 4 Implementation pathways: systematic restructuring from curriculum to assessment

### 4.1 Curriculum integration pathways

To restructure nursing education around public health literacy, curriculum integration should follow two main approaches (Table 1). First, systematically embed public health cases into existing clinical courses (34). For example, when teaching diabetes management in medical-surgical nursing, instruction should go beyond individual glycemic control and medication adherence. Students should also learn to analyze available community health resources, patients’ social support networks, and the social determinants that influence treatment adherence (10). Through such case designs, students gradually develop the ability to combine clinical problem-solving with a population health perspective (10). Second, add interdisciplinary modules on “Nursing and Public Health” (9, 15). These modules may cover infectious disease outbreak investigation, health policy analysis, and health communication strategies. Together, these two approaches complement each other and promote the systematic integration of public health literacy into theoretical teaching.

**Table 1:** Key components and implementation strategies of public health literacy in nursing education.

Domain	Key elements	Educational strategies	Expected outcomes
Core competencies	Epidemiological thinking, health promotion, etc.	Integrated teaching, case-based learning	Population health perspective
Curriculum	Public health embedded courses	Interdisciplinary modules	Knowledge integration
Practice	Community-based learning	Service-learning projects	Real-world competence
Assessment	Multidimensional evaluation	OSCE+project-based assessment	Comprehensive literacy
Faculty	Public health training	Interdiscipalinary collaboration	Teaching capacity
Policy/System	Accreditation integration	Institutional collaboration	Sustainable reform

OSCE, objective structured clinical examination.

### 4.2 Practice-based teaching pathways

Practice-based teaching is essential for internalizing public health literacy (35). First, implement community-based learning by extending students’ practice settings from hospitals into community environments (9). Specific activities include home visits, participation in school health projects, and health promotion programs for migrant populations (9). In these real-world contexts, students should identify population health risks, communicate effectively with community members, and design interventions suited to local conditions (14). Second, adopt a service-learning integration model, wherein nursing students collaborate with local public health departments to deliver services such as vaccination campaigns, health screenings, and health education (36). This model not only strengthens students’ sense of social responsibility but also allows them to practice interprofessional collaboration and population health management through real-world tasks (36, 37).

### 4.3 Assessment system pathways

Traditional assessments of nursing skills cannot adequately capture public health literacy; therefore, the assessment system requires restructuring (38). First, develop a dedicated public health literacy scale to measure students across multiple dimensions, including knowledge, attitudes, and behaviors (38). Second, add community health scenarios—such as simulated home visits or community needs assessments—to the objective structured clinical examination to evaluate students’ judgment and communication skills in non-clinical settings (39). Third, introduce project-based assessment, requiring students to write a community health improvement plan that includes problem identification, goal setting, intervention strategies, and outcome evaluation (39, 40). This approach more authentically captures students’ ability to translate public health knowledge into actionable plans.

### 4.4 Faculty and institutional pathways

Effective implementation of this systematic restructuring depends on faculty development and institutional support. Regarding faculty, provide interdisciplinary training to help nursing educators systematically learn core public health knowledge—including epidemiology, social medicine, and health promotion—thereby strengthening their ability to incorporate a public health perspective into their teaching (41, 42). Regarding institutions, establish joint training mechanisms between nursing schools and schools of public health (9). These may include co-supervision by faculty from both disciplines, shared curriculum resources, and mutual credit recognition. Through institutionalized cross-school collaboration, the sustainability and academic rigor of nursing education restructuring can be ensured (14, 25).

## 5 Challenges, strategies, and future research directions

### 5.1 Potential barriers to implementation

Integrating public health literacy into nursing education may face several barriers. First, curriculum density is a major concern (9). Current nursing curricula are already full, covering basic medicine, clinical nursing, and social sciences (9). Adding public health content may overload students and compete for limited teaching time (9). Second, faculty structure is another barrier. Most nursing educators have clinical backgrounds but lack public health training, making it difficult for them to teach epidemiology, social medicine, or health policy (14). Third, assessment system inertia also poses a problem (15). Existing assessments focus on individual clinical skills, creating path dependence among faculty and students (15). Moving toward multidimensional assessments that include population health will take time. These barriers are interconnected and should be considered early in any reform effort.

### 5.2 Response strategies

Three strategies can address these challenges. First, strengthen policy guidance. Education and health authorities should include public health literacy in nursing accreditation standards and licensure exams, using policy to drive change (41). Second, develop demonstration courses. Support selected institutions to create integrated “Nursing and Public Health” courses, along with shareable teaching cases, textbooks, and assessment tools (43, 44). This will lower the cost of reform for other schools. Third, learn from international experience. For example, some US nursing schools require students to complete community health projects and show population health skills before graduation (14, 45). Such approaches should be adapted to local contexts, not copied directly.

In this context, aligning nursing education reform with established international competency frameworks can further accelerate implementation and enhance coherence (1). The American Association of Colleges of Nursing Essentials framework, widely adopted across US nursing programs, provides a structured and competency-oriented approach for integrating population health into nursing education (30, 31). Its incorporation into curriculum design can promote standardization, facilitate cross-institutional comparability, and improve global applicability (46). Similarly, competency toolkits developed by the Consortium of Universities for Global Health offer actionable guidance for embedding interprofessional collaboration and population-based perspectives into training systems (32). Together, these frameworks serve as practical reference models that can support the systematic translation of reform strategies into educational practice (46).

Collectively, these strategies provide a coordinated pathway for overcoming structural barriers and advancing the integration of public health literacy into nursing education systems (1).

### 5.3 Future research directions

Future research should pursue three directions. First, study how public health literacy affects nursing graduates’ work outcomes (47). Researchers could track graduates’ job performance in clinical or community settings and examine links between their literacy levels and patient health outcomes or community service capacity (47). Second, explore different educational models for various training stages. Undergraduate programs should focus on basic public health knowledge and attitudes. Master’s programs should emphasize program design and evaluation. Continuing education should support role transition and skill enhancement for practicing nurses. Third, investigate the mediating mechanisms between public health literacy and health equity (48). For instance, do nurses’ community assessment skills and policy sensitivity reduce health disparities by improving resource allocation or service access? These studies will provide evidence to guide ongoing nursing education reform.

## 6 Summary

This perspective article has argued that contemporary public health challenges—including infectious disease pandemics, rising chronic disease burdens, and persistent health inequities—require a fundamental reconceptualization of nursing education. The proposed theoretical framework positions public health literacy as a core competency. It integrates epidemiological thinking, health promotion, community engagement, policy sensitivity, and cultural safety across curricula, practice, assessment, and institutional support systems. Unlike traditional models that prioritize individual clinical skills, this framework treats public health literacy not as an add-on but as a transformative element that reshapes professional nursing identity. Achieving this vision requires coordinated action. Policymakers should embed public health literacy into accreditation and licensure standards. Educational institutions should redesign curricula and foster cross-disciplinary faculty development. Funding agencies need to support demonstration projects and evaluative research. Only through such systemic collaboration can nursing education prepare a workforce capable of advancing both clinical excellence and population health in an increasingly complex public health landscape.

## Data Availability

The original contributions presented in the study are included in the article/supplementary material, further inquiries can be directed to the corresponding author.
